# Venetoclax concentration affects the incidence of haematological adverse events in patients with acute myeloid leukaemia

**DOI:** 10.3389/fphar.2025.1710282

**Published:** 2026-01-07

**Authors:** Jing Chen, Xi Yang, Jingxian Xie, Lijuan Zhang, Lu Chen

**Affiliations:** 1 Department of Pharmacy, Guangyuan Central Hospital of Sichuan Province, Guangyuan, China; 2 School of Medicine, University of Electronic Science and Technology of China, Department of Hematology, Sichuan Provincial People’s Hospital, Sichuan Academy of Medical Sciences, Chengdu, China; 3 Department of Pharmacy, Personalized Drug Therapy Key Laboratory of Sichuan Province, Sichuan Academy of Medical Sciences and Sichuan Provincial People’s Hospital, School of Medicine, University of Electronic Science and Technology of China, Chengdu, China; 4 School of Pharmacy, Southwest Medical University, Luzhou, Sichuan, China; 5 Department of Pharmacy, Pujiang People’s Hospital, Chengdu, China

**Keywords:** venetoclax, concentration, combination medication, CYP3A inhibitors, haematological adverse events

## Abstract

**Introduction:**

Venetoclax (VEN) demonstrates considerable inter-individual variability in drug exposure, and its pharmacokinetics are substantially influenced by concurrent administration of CYP3A inhibitors. This study explores the relationship between VEN exposure and the incidence of haematological adverse events (AEs) in patients by quantifying systemic VEN concentrations.

**Methods:**

We retrospectively analyzed data from 114 patients who received VEN therapy. Among these, 52 were treated with VEN combined with azacitidine (AZA, days 1–7), 20 received VEN + AZA combined with omeprazole (OPZ), 20 received VEN + AZA combined with voriconazole (VCZ), and 22 received VEN + AZA combined with posaconazole (PCZ). High-performance liquid chromatography was used to determine the plasma steady-state C_min_ and C_max_ of VEN (n = 114), and the plasma steady-state C_min_ of VCZ (n = 20) and PCZ (n = 22).

**Results:**

The effect of combined medication on VEN concentration was as follows: VEN + PCZ group/VEN + VCZ group > VEN + OPZ group > VEN group. Increased VEN concentration increased the incidence of Grade 3 or higher haematological AEs. CYP3A inhibitors increased VEN levels, thereby increasing the risk of AEs. A VEN C_max_ value of 4,886.00 ng/mL had the highest predictive value for mitigating AEs in patients presenting with Grade ≥3 neutropenia and thrombocytopenia.

**Conclusion:**

VEN concentration is correlated with the occurrence of haematological AEs, and VEN concentrations can be used to predict the occurrence of haematological AEs.

## Graphical Abstract

**Figure:**
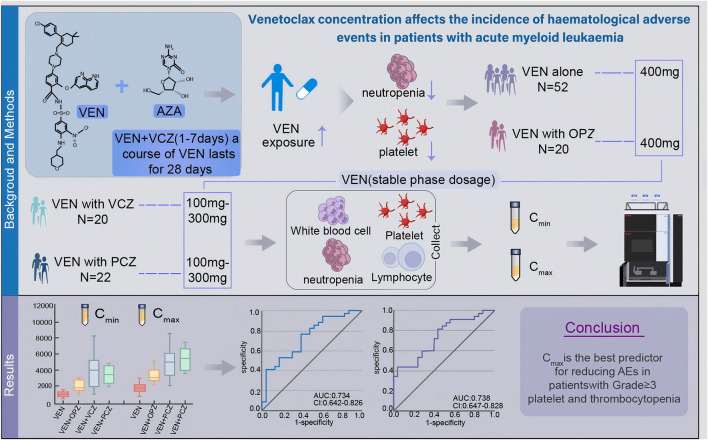

## 1 Introduction

Venetoclax (VEN) is a selective B-cell lymphoma-2 (BCL-2) inhibitor. It binds to Bcl-2 and displaces pro-apoptotic proteins, initiating an apoptotic cascade of malignant cells (DiNardo et al., 2019). VEN has been approved for treating chronic lymphocytic leukaemia, acute myeloid leukaemia (AML), and multiple myeloma (Gong et al., 2023; Bennett and Seymour, 2024; Kumar et al., 2023). VEN, combined with azacitidine (AZA) or low-dose cytarabine, is the standard treatment for AML patients unfit for intensive chemotherapy (Turkalj et al., 2023) owing to its high selectivity and low haematological toxicity (Juárez-Salcedo et al., 2019; Leukemia and Lymphoma Group, Chinese Society Of Hematology, Chinese Medical Association, 2023b).

Increased exposure to VEN may lead to life-threatening tumour lysis syndrome (TLS, 12%), with common adverse events (AEs) such as neutropenia (NEUT) (45%), diarrhoea (35%), nausea (33%), anaemia (29%), low platelet count (PLT) (22%) (Al-Sawaf et al., 2020; Brackman et al., 2022). The prescribing information for VEN stipulates that treatment should not be interrupted due to cytopenia in patients with AML until remission is achieved. However, data from the VIALE-A trial, which evaluated the combination of VEN and AZA, indicate that most patients require dose adjustments of VEN to effectively manage cytopenia (Vachhani et al., 2022). Population pharmacokinetic studies of VEN demonstrated that mild to moderate renal and hepatic impairment, body weight, and age had no effect on the pharmacokinetics of VEN (Jones et al., 2016). Two main factors influence VEN dosage adjustment: the risk of treatment-related toxicity, such as decreased blood cell counts, and potential drug interactions, especially with concurrent CYP3A inhibitors. Co-administration of VEN with the potent CYP3A inhibitor posaconazole (PCZ) and the moderate inhibitor voriconazole (VCZ) significantly elevated VEN exposure (Agarwal et al., 2017; Dong et al., 2022). Omeprazole (OPZ) is commonly used to prevent or reduce gastrointestinal reactions in patients with hematologic malignancies undergoing chemotherapy (Wang et al., 2021). As a CYP3A enzyme inhibitor, OPZ may interact with VEN, causing drug-drug interactions (DDIs).

In this study, VEN plasma concentrations were subjected to therapeutic drug monitoring (TDM) to evaluate the resulting effects when used in combination with VCZ, PCZ, and OPZ and to evaluate the correlations between changes in VEN exposure and haematological AEs.

## 2 Patients and methods

### 2.1 Study design

This clinical study was approved by the Ethics Committee of the Sichuan Provincial People’s Hospital (No. 2023-102). This study was a retrospective observational study; thus, no informed consent was required. Included AML patients intolerant to intensive chemotherapy from March to December 2023, excluding those with acute promyelocytic leukemia. AML diagnosis was based on the 2016 WHO classification of hematopoietic and lymphoid tumors. Patients received a low-intensity regimen of VEN combined with AZA, with no additional chemotherapy treatments administered. Patients received VEN treatment for 28 days. VEN was administered with a dose-escalation period of 1–3 days, at doses of 100 mg, 200 mg, and 400 mg; from the fourth day until the end of the treatment cycle, the dose remained 400 mg. When VEN was used in combination with VCZ or PCZ, the dose of VEN was reduced, with the actual usage ranging from 100 to 400 mg. From day 1–7 of the treatment course, VEN was used in combination with AZA, which was given administered subcutaneously at a dose of 75 mg/m^2^. Given AZA’s adverse effects, such as cytopenia and leukopenia, blood cell counts should return to baseline after treatment. Then, a stable VEN dose should be maintained for at least 4 days to monitor its concentration. The patients were administered VCZ and PCZ for prophylaxis against invasive aspergillosis, with the doses determined by the attending physician.

### 2.2 Study objectives

The main outcomes measured in this study were the association between changes in VEN concentration and Grade ≥3 haematological AEs, including those related to white blood cell count (WBC), NEUT, lymphocyte count (LY), and PLT, as well as the use of VEN drug concentrations were also used to predict haematological AEs. Secondary outcomes included the impact of combination CYP3A inhibitors on VEN concentration in clinical practice and individualised dose adjustments for patients based on their monitored VEN concentration results. AE grading was in accordance with the Common Toxicity Criteria of the National Cancer Institute (version 5.0).

### 2.3 VEN and VCZ/PCZ assays

The concentration of VEN was quantified utilising a methodologically-validated high-performance liquid chromatography technique. The minimum (C_min_) and maximum (C_max_) mentioned both refer to the steady-state concentrations. The C_min_ was determined 0.5 h before drug administration, and C_max_ was collected 6 h after taking VEN, with all samples were stored at −20 °C until analysis. VEN exhibited a good linear relationship with the peak area within the concentration range of 200.00–12000.00 ng/mL. The regression equation of the standard curve was Y = 0.3269X + 0.0158 (r^2^ = 0.999), and the lower limit of quantitation was 200.00 ng/mL. The C_min_ concentrations of VCZ and PCZ were assessed on day 5 and 7 following administration, espectively, with blood samples collected 0.5 h before drug administration. Concentration monitoring of VCZ/PCZ was conducted as part of an existing treatment drug monitoring project at this hospital, using a validated methodology.

### 2.4 Statistical analysis

Normally distributed data are expressed as mean ± standard deviation ( $\bar{x}$ ± s). The two groups were compared using *t*-tests for both homogeneous and unequal variances. If the data were not normally distributed, the median and quartiles are reported as [M (Q1, Q3)]. The Mann–Whitney U test was used to compare two groups, and the Kruskal–Wallis H test was used to compare multiple groups. Statistical analysis was performed using SPSS 27.0, with the cutoff for statistical significance set at P < 0.05.

## 3 Results

### 3.1 Patient characteristics

Patients who did not undergo routine blood tests within 3 days before or after VEN concentration detection, those with significantly abnormal blood samples such as plasma deterioration or haemolysis, or those with severe liver impairment (Child-Pugh C; these patients themselves require VEN dosage adjustment) were excluded.

**FIGURE 1:**
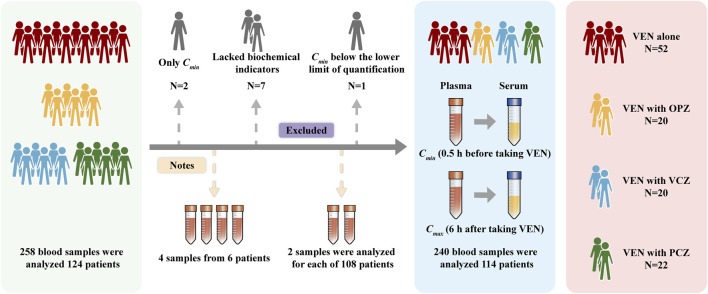
Patient inclusion status diagram.

The concentration monitoring results included VEN concentrations from 114 patients, VCZ concentrations from 20 patients, and PCZ concentrations from 22 patients. Of the 114 patients, 60 were male and 54 were female, aged 16–82 years (average: 57.09). None smoked or consumed alcohol. All patients received a standard treatment regimen, with VEN + AZA administered from days 1–7. After 7 days, 52 patients received VEN (400 mg, QD) treatment alone, while 20 patients received VEN (400 mg, QD) in combination with intravenous OPZ. Additionally, 20 patients received VCZ treatment (14 via intravenous injection, 6 orally) while being treated with VEN (100–300 mg, QD). Furthermore, 22 patients were orally administered a PCZ suspension while also receiving VEN (100–300 mg, QD). The result is presented in Figure 1.

### 3.2 VEN concentration

Patients were classified into VEN (Group 1), VEN + OPZ (Group 2), VEN + VCZ (Group 3), and VEN + PCZ (Group 4) based on their medication status. Group 1 included 52 patients who received VEN at a stable dose of 400 mg/d. The patients’ C_min_ and C_max_ concentration ranges were 365.70–2,738.60 ng/mL and 695.90–2,999.40 ng/mL, respectively. In total of 20 patients received VEN combined with OPZ (Group 2), the stable dose of VEN at 400 mg/d, C_min_ ranged 837.90–4,734.40 ng/mL for and C_max_ ranged 2,154.40–7,886.50 ng/mL. In Group 3, 10 patients received VEN with VCZ at 100 mg/d, 8 at 200 mg/d, and 2 at 300 mg/d. The C_min_ and C_max_ concentration ranges were 969.40–8,439.30 ng/mL and 1,569.00–8,654.50 ng/mL, respectively. In Group 4, among the patients receiving VEN with PCZ, 10, 10, and 2 received 100 mg/d, 200 mg/d, and 400 mg/d, respectively. The patients’ C_min_ and C_max_ concentration ranges were 1923.00–10856.10 ng/mL and 3,571.10–11231.70 ng/mL, respectively.

The concentration ranges for the 20 and 22 patients receiving VCZ and PCZ were 0.23–5.17 μg/mL [reference: 1–5.5 μg/mL (Ashbee et al., 2014)] and 0.28–1.29 μg/mL [reference: ≥0.7 μg/mL (Ashbee et al., 2014)], respectively. The VCZ/PCZ concentrations of patients treated for therapeutic purposes were within the reference ranges. In some patients, VCZ/PCZ concentrations were not within the reference ranges, and these patients were treated with a combination of VCZ/PCZ and VEN to reduce the cost of VEN treatment. See Table 1 for details.

**TABLE 1:** Concentration detection results.

Goup	VEN/C_min_ ng/mL	VEN/C_max_ ng/mL	VCZ/PCZ C_min_ (μg/mL)
1	903.85 (668.23, 1,108.48)	1855.25 (1,201.53, 1961.08)	
2	1794.40 (1,511.63, 2,713.88)	3,272.60 (2,910.83, 3,931.38)	
3	3,938.23 ± 2,169.93	4,889.12 ± 2093.68	2.26 ± 1.49
4	3,409.05 (2,417.18, 4,588.03)	5,319.45 (4,034.78, 6,684.03)	0.78 ± 0.36

Abbreviations: VEN, venetoclax; C_min_, minimum; C_max_, maximum; VCZ, voriconazole; PCZ, posaconazole.

### 3.3 Effect of combined medication on VEN levels

**FIGURE 2:**
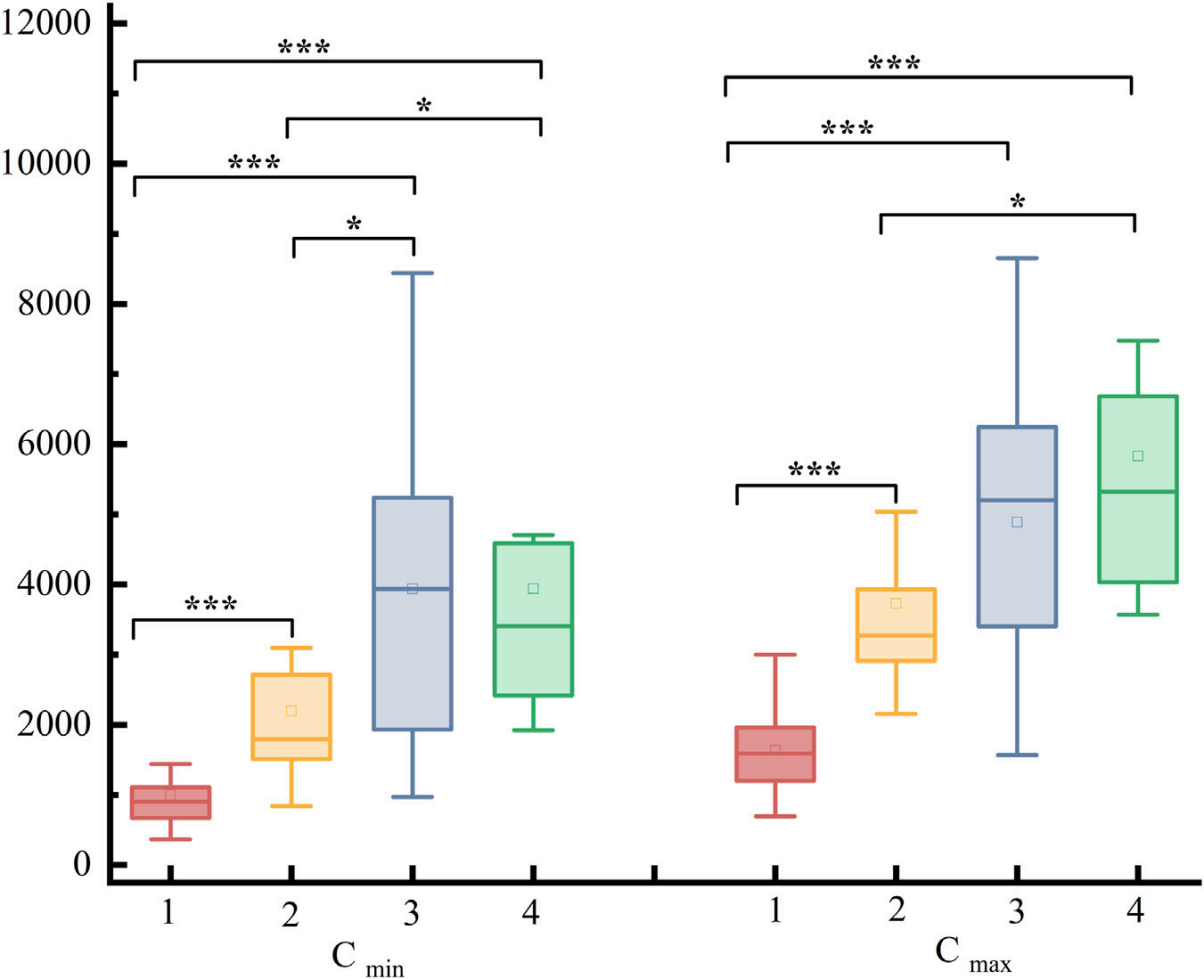
Distribution of C_min_ and C_max_ among the groups and their differences.

The combination of OPZ, VCZ, and PCZ with VEN increased VEN concentrations compared with VEN alone (P < 0.05). Differences in C_min_ and C_max_ between Group 1 and Groups 2, 3, and 4 (P < 0.001), differences in C_min_ between Groups 2 and 3 (P < 0.05), and differences in C_min_ and C_max_ between Groups 2 and 4 (P < 0.05). No statistically significant C_min_ and C_max_ differences were found in C_min_ and C_max_ between Groups 3 and 4. These results are presented in Table 2; Figure 2.

**TABLE 2:** Differences in VEN’s C_min_ and C_max_ among groups.

Go-up	C_min/_ng/mL	Z/t	P	C_max/_ng/mL	Z/t	P
1 2	903.85 (668.23, 1,108.48)1794.40 (1,511.63, 2,713.88)	−4.703	<0.001***	1855.25 (1,201.53, 1961.08) 3,272.60 (2,910.83, 3,931.38)	−5.646	<0.001***
1 3	903.85 (668.23, 1,108.48)3,938.23 ± 2,169.93	−5.985	<0.001***	1855.25 (1,201.53, 1961.08) 4,889.12 ± 2093.68	−6.859	<0.001***
1 4	903.85 (668.23, 1,108.48)3,409.05 (2,417.18, 4,588.03)	−5.649	<0.001***	1855.25 (1,201.53, 1961.08) 5,319.45 (4,034.78, 6,684.03)	−9.101	<0.001***
2 3	1794.40 (1,511.63, 2,713.88) 3,938.23 ± 2,169.93	−3.211	0.003*	3,272.60 (2,910.83, 3,931.38) 4,889.12 ± 2093.68	−1.956	0.058
2 4	1794.40 (1,511.63, 2,713.88) 3,409.05 (2,417.18, 4,588.03)	−2.966	0.005*	3,272.60 (2,910.83, 3,931.38) 5,319.45 (4,034.78, 6,684.03)	−3.562	<0.001***
3 4	3,938.23 ± 2,169.93 3,409.05 (2,417.18, 4,588.03)	−0.005	0.996	4,889.12 ± 2093.68 5,319.45 (4,034.78, 6,684.03)	−1.440	0.158

Abbreviations: VEN, venetoclax; OPZ, omeprazole; VCZ, voriconazole; PCZ, posaconazole; C_min_, minimum; C_max_, maximum.

The VEN dose was adjusted for six patients: two each of the VEN + VCZ, VEN + PCZ, and VEN + OPZ groups. The concentrations in two patients from the VEN + OPZ group decreased after replacing OPZ with rabeprazole, enabling VEN to reach the therapeutic window. The VEN dosage for the remaining four patients was adjusted based on the therapeutic window of C_min_ (500–4,000 ng/mL) and C_max_ (2000–5,500 ng/mL) (Yang et al., 2022; Brackman et al., 2022; Agarwal et al., 2017). After adjusting the VEN dosage, these patients reached the therapeutic window, leading to the recovery of PLT (reference: 125–350 × 10^9^/L), WBC (reference: 3.5–9.5 × 10^9^/L), NEUT (reference: 1.8–6.3 × 10^9^/L), and LY (reference: 1.1–3.2 × 10^9^/L) levels. See Table 3 for details.

**TABLE 3:** Biochemical indicator changes before and after VEN dose adjustment.

Gro-up	N	Time	VEN (mg)	C_min_ ng/mL	C_max_ ng/mL	WBC 10^9^/L	NEUT 10^9^/L	LY 10^9^/L	PLT 10^9^/L
3	1	Before	200	4,200.00	5,481.00	0.48	0.08	0.4	28
		After	100	2023.31	2,418.26	0.61	0.16	0.43	56
	2	Before	200	5,234.40	5,355.70	1.1	0.42	0.57	15
		After	100	1,478.40	1847.31	1.41	0.59	0.55	20
4	1	Before	400	10,856.00	11,231.60	0.2	0.09	0.1	17
		After	100	2089.14	2,449.34	1.8	1.06	0.51	46
	2	Before	200	4,706.00	7,473.40	0.89	0.33	0.16	98
		After	100	1923.14	2,911.22	2.08	1.92	0.55	102

Abbreviations: VEN, venetoclax; C_min_, minimum; C_max_, maximum; WBC, white blood count; NEUT, neutrophil count; LY, lymphocyte count; PLT, platelet count.

### 3.4 Impact of VEN concentration on haematological AEs

Ninety-four patients had Grade ≥3 haematological AEs on VEN. The incidence rates of grade 3 and above WBC reduction AEs in different groups were 53.84% (Group 1), 40.00% (Group 2), 90.00% (Group 3), and 72.73% (Group 4), respectively. The incidence rates for NEUT reduction were 50.00% (Group 1), 40.00% (Group 2), 90.00% (Group 3), and 81.82% (Group 4)The incidence rates for LY reduction were 42.31% (Group 1), 20.00% (Group 2), 70.00% (Group 3), and 72.73% (Group 4). The incidence rates for PLT reduction were 50.00% (Group 1), 60.00% (Group 2), 80.00% (Group 3), and 63.64% (Group 4). The incidence of Grade ≥3 haematological AEs in Group 2 was no higher than that of Group 1, but there was a significant rise in AEs in Groups 3 and 4. These trends are consistent with the concentration changes observed across these groups.

Patients with Grade ≥3 haematological AEs were classified as experiencing AEs, and those without were classified into the N-AE category. The VEN C_min_ was higher in the group with AEs of Grade ≥3 for WBC, NEUT, LY, and PLT compared to the group without (P < 0.05). The VEN C_max_ was also higher in the group with Grade ≥3 adverse events for NEUT, LY, and PLT (P < 0.05). See Table 4 for details.

**TABLE 4:** C_min_ and C_max_ differences in patients with haematological AEs.

AE type	Group	C_min_/ng/mL	Z/t	P	C_max_/ng/mL	Z/t	P
WBC	AE	1896.65 (969.48, 3,674.93)	2.555	0.066	2,999.35 (1,599.58, 5,354.73)	1.857	0.012*
	N-AE	1,328.70 (893.63, 2,434.78)			2,459.15 (1,519.23, 3,931.38)		
NEUT	AE	2,417.10 (1,067.00, 3,675.00)	4.026	<0.001***	3,571.10 (2092.80, 5,481.10)	4.413	<0.001***
	N-AE	1,108.40 (668.30, 1931.50)			1742.60 (1,224.00, 3,310.50)		
LY	AE	2,440.55 (1,079.33, 3,674.98)	2.587	0.048*	3,301.00 (1834.23, 5,162.48)	1.997	0.012*
	N-AE	1,418.85 (837.98, 2,434.73)			2,154.45 (1,540.28, 4,260.73)		
PLT	AE	2,550.60 (1,070.08, 4,179.23)	4.493	<0.001***	3,362.25 (2028.83, 6,266.23)	4.857	<0.001***
	N-AE	1,108.45 (813.05, 1999.55)			1742.55 (1,437.58, 3,400.95)		

Abbreviations: C_min_, minimum; C_max_, maximum; WBC, white blood count; NEUT, neutrophil count; LY, lymphocyte count; PLT, platelet count.

**FIGURE 3:**
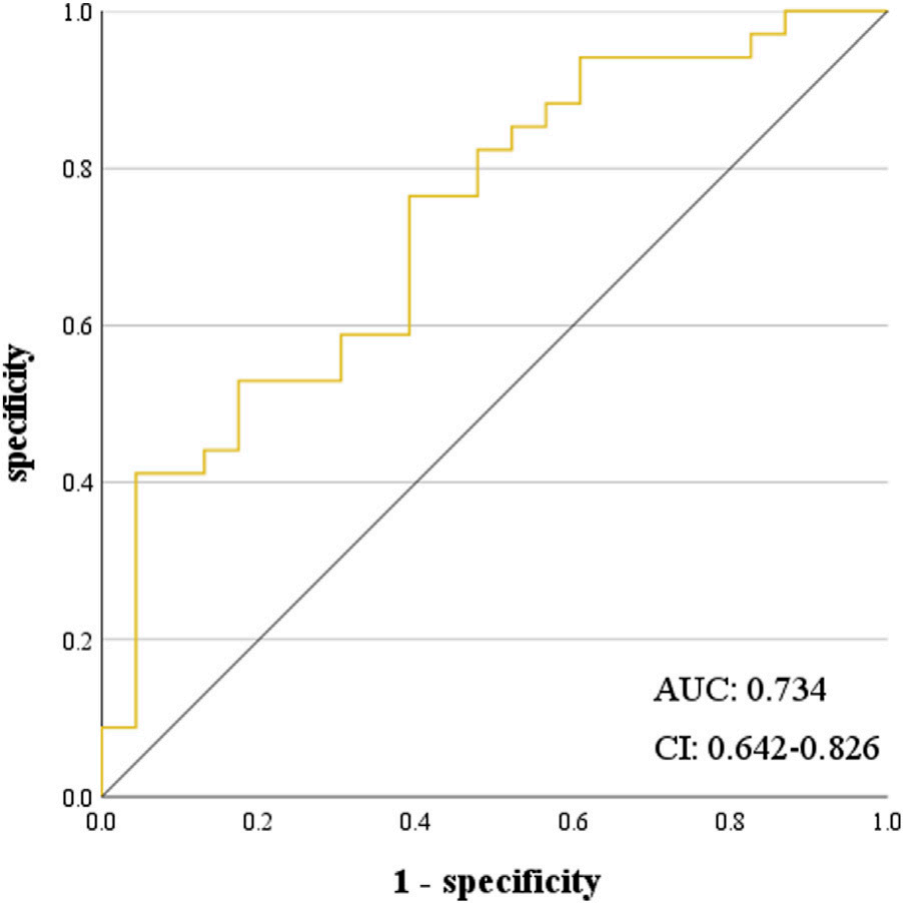
ROC curve of C_max_ predicting ≥3 grade NEUT reduction.

**FIGURE 4:**
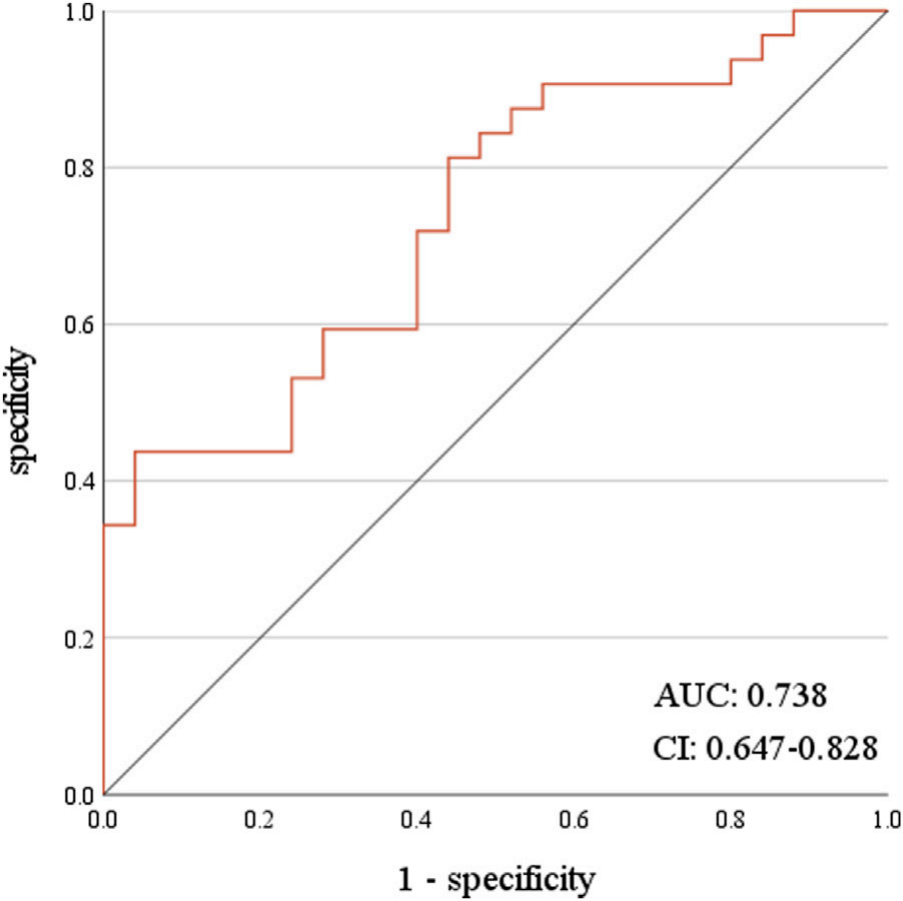
ROC curve of C_max_ predicting ≥3 grade PLT reduction.

Regarding the effectiveness of VEN concentration in predicting haematological AEs, VEN C_max_ was the best predictor for Grade ≥3 PLT and NEUT AEs. At a C_max_ value of 4,886.00 ng/mL, the sensitivity was 41.80%, and the specificity was 95.70%. The receiver-operating characteristic (ROC) curve area was 0.734, showing that predicting Grade ≥3 NEUT reduction-related AEs based on the C_max_ of VEN had a performance rate of 73.40% (P < 0.001, 95% CI: 0.642–0.826) (Figure 3). At a C_max_ value of 4,886.00 ng/mL, the sensitivity was 43.80%, and the specificity was 96.00%. The ROC curve area was 0.738, showing that predicting Grade ≥3 PLT reduction AEs based on the C_max_ of VEN had a performance rate of 73.80% (P < 0.001, 95% CI: 0.647–0.828) (Figure 4).

## 4 Discussion

VEN was granted FDA breakthrough therapy designation and accelerated approval, along with the EU orphan drug designation for multiple myeloma, diffuse large B-cell lymphoma, and other non-Hodgkin lymphomas (Salem and Menon, 2024). VEN is the first-line treatment for AML, relapsed and refractory AML, and chronic lymphocytic leukaemia/small lymphocytic lymphoma in multiple guidelines and countries (Hematology Committee of Chinese Medical Association, Hematological Oncology Committee of China Anti-Cancer Association, 2022; Leukemia and Lymphoma Group, Chinese Society of Hematology, Chinese Medical Association, 2023a; Leukemia and Lymphoma Group, Chinese Society Of Hematology, Chinese Medical Association, 2023b). This retrospective observational study analysed the TDM of VEN in patients with haematological tumours. It evaluated the effects of concomitant medications on VEN concentration, the relationship between VEN concentration changes and AEs, and the association between combined medications and concentration-related AEs.

### 4.1 VEN TDM

VEN levels vary widely among individuals and moderate or strong CYP3A inhibitors can significantly affect drug exposure (Gong et al., 2023). In this study, patients in the VEN-only group had a steady-state C_min_ of 969.80 (699.00, 1,150.60) ng/mL and a C_max_ of 1719.03 ± 541.85 ng/mL after receiving VEN 400 mg/d. The average steady-state C_min_ and C_max_ values for Chinese patients on VEN 400 mg/d are 1,020.00 ± 730.00 ng/mL and 2,970.00 ± 1,590.00 ng/mL, respectively (Yang et al., 2022). The C_max_ for Japanese patients on VEN 400 mg/d is 2080 ± 1,040 ng/mL (Izutsu et al., 2020). The C_min_ results matched those in the literature, but there were significant differences in the C_max_ data. Studies have shown that consuming VEN with food increases its absorption, with low-fat and high-fat diets affecting C_max_ differently (Salem et al., 2016). Meal management of enrolled patients differs between this study and other reported studies (Yang et al., 2022; Izutsu et al., 2020), affecting VEN bioavailability and resulting in differences in reported C_max_ values. Existing literature reports that polymorphisms in the CYP3A and ABCB1 genes may affect VEN plasma concentration. A Finnish study reported extremely high VEN concentrations in a patient homozygous for the CYP3A4*22 c.522-191G>A variant (one case), indicating this SNP may affect VEN pharmacokinetics (Kytölä et al., 2025). A Chinese study suggests that patients with GG CYP3A5 rs776746 and TT/CT ABCB1 rs1045642 may have elevated VEN levels (Li et al., 2024). Genetic polymorphism may also be a reason for the significant individual differences in VEN concentration. Given that VEN levels are influenced by various factors, conducting TDM for VEN is essential.

### 4.2 Combined effects of OPZ and VEN

Two patients in this study were treated with VEN and OPZ. Adjusting OPZ to non-enzyme-metabolised rabeprazole while keeping the VEN dosage constant resulted in a significant decrease in VEN concentration. In patients administered OPZ, the VEN concentration significantly increased, however, OPZ’s effect on gastric acid pH did not impact VEN bioavailability or absorption rate (Jones et al., 2016). Moderate and strong CYP3A inhibitors decreased venetoclax apparent clearance by 19% and 84%, respectively, while weak CYP3A inhibitors and inducers did not affect clearance (Jones et al., 2016). OPZ is neither a moderate nor a strong CYP3A inhibitor. OPZ does not seem to influence the VEN clearance phase. OPZ acts as both a substrate and inhibitor of the CYP2C19 and CYP3A4 enzymes (Moreau et al., 2006; Takahashi et al., 2007). CYP2C19 primarily mediates OPZ’s 5-hydroxylation metabolism. However, in individuals with CYP2C19 gene mutations (poor metabolizers), once CYP2C19 is saturated, CYP3A4 becomes the primary enzyme metabolizing OPZ (Rost and Roots, 1996). Studies indicate that tacrolimus (metabolized by CYP3A4 and CYP3A5) and omeprazole may compete for CYP3A4, potentially increasing tacrolimus levels (Miedziaszczyk and Idasiak-Piechocka, 2023). Although OPZ is not a moderate or strong CYP3A inhibitor, VEN and OPZ may compete for CYP3A4 enzyme activity, potentially increasing VEN levels, particularly in patients with CYP2C19 gene mutations who are poor metabolizers.

### 4.3 Combined effects of PCZ, VCZ, and VEN

The interaction between triazole antifungals and VEN has gained attention in clinical treatment (Stemler et al., 2022; Lewis et al., 2024). Although VCZ and PCZ are both CYP3A inhibitors, PCZ has a stronger inhibitory effect than VCZ (Chau et al., 2014; Luo et al., 2019); PCZ affects VEN concentration more than VCZ. No significant differences in C_min_ and C_max_ were found between the VEN + VCZ and VEN + PCZ groups in this study, likely because of the different administration routes used: intravenous for VCZ and oral suspension for PCZ. All patients received PCZ suspension. The high lipophilicity and low water solubility of PCZ lead to low and unstable oral bioavailability (Krishna et al., 2009). Injections and enteric-coated tablets offer greater average concentrations and drug exposure than suspensions (Krishna et al., 2012; Salmanton-Garcia et al., 2019). The bioavailability gap between the different dosage forms may account for the lack of significant differences in C _min_ and C_max_ between the VEN + VCZ and VEN + PCZ groups.

### 4.4 Drug combinations and VEN concentration-related AEs

AEs such as decreased NEUT and PLT are common during VEN treatment. Studies have shown that 7.4% of patients discontinue VEN due to AEs, while some experience acute, fatal AEs (Roeker et al., 2019; Jie et al., 2023). VEN exposure is correlated with NEUT in reducing AEs (Brackman et al., 2022). Research has shown that combining VEN with triazole drugs significantly increases the median recovery time of NEUT compared to that of the VEN-only treatment (Roeker et al., 2019; Rausch et al., 2021). This study found that Grade ≥3 haematological AEs were significantly more common in the VEN + VCZ and VEN + PCZ groups than in the VEN-only group. In contrast, the concentration of VEN increased with VEN + OPZ treatment, but the incidence of Grade 3 haematological AEs in the VEN group did not. OPZ was associated with lower rates of haematological AEs; however, haematological AEs caused by VCZ and PCZ treatment were more common. Therefore, When VEN is combined with VCZ and PCZ, hematologic adverse events may accumulate in patients. Adjusting the VEN dosage for six patients reached the target drug concentration, resulting in the recovery of PLT, WBC, NEUT, and LY levels. This indicates that higher VEN concentrations may elevate the incidence of Grade ≥3 AEs.

## 5 Conclusion

The concomitant use of CYP3A inhibitors increased VEN exposure and raised the incidence of Grade ≥3 haematological AEs. A VEN C_max_ value of 4,886.00 ng·mL^-−1^, demonstrates the highest predictive value for Grade ≥3 NEUT and PLT AEs in patients. Consequently, clinicians must closely monitor alterations in VEN concentrations to prevent AEs or potential drug discontinuation resulting from elevated VEN concentrations, particularly when it is used in conjunction with CYP3A inhibitors.

This study has several limitations, including a small sample size, a relatively short follow-up period, and the omission of clinical efficacy indicators. These factors underscore the need for further research to ensure clinical relevance and effectiveness. A more precise application of VEN along with the exploration of combination therapies will yield additional evidence to support the objectives of rational medication use and personalised treatment strategies.

## Data Availability

The original contributions presented in the study are included in the article/Supplementary Material, further inquiries can be directed to the corresponding authors.
